# Should Patients With Autism-Related Restricted Eating Have Long-Term Enteral Feeding? A Case Study

**DOI:** 10.1192/bjo.2025.10784

**Published:** 2025-06-20

**Authors:** Heidi Turner

**Affiliations:** Dartford and Gravesham NHS Trust, Dartford, United Kingdom

## Abstract

**Aims:** Avoidant Restrictive Intake Disorder (ARFID) is characterised by insufficient intake, for reasons unrelated to body image concerns, but is strongly associated with autism spectrum disorder (ASD). It causes weight loss, nutritional deficiencies and physical health consequences, which can be fatal, as seen in the tragic 2021 case of Alfie Nicholls. Despite ARFID’s impact, there are no national guidelines and treatment recommendations are limited, advising psychological interventions and nutritional counselling.

**Methods:** A male in his thirties with ASD and restricted eating, resulting in multiple admissions for malnutrition, including to intensive care, was readmitted with electrolyte disturbance and dehydration. Numerous psychiatric assessments concluded his restrictive eating to be ASD-associated ARFID. Despite psychological and nutritional support throughout his life, he was unable to maintain a healthy BMI and developed chronic malnutrition. Similarly, during this admission after four months of dietetic input, nasogastric feeding, which he found difficult to tolerate, and psychological intervention, he lost weight, causing recurrent infections, persistent anaemia and perforated gallbladder secondary to gallstones. Long-term enteral feeding (LEF) had not previously been explored, so there were multi-disciplinary team discussions, deciding a percutaneous endoscopic gastrostomy (PEG) would be in his best interests. This enabled him to reach a safe weight for discharge, continue to gain weight, improve his general health and minimise readmission risk.

**Results:** Decision-making is challenging due to a lack of research on ARFID management and the role of LEF. The treatments currently recommended are sometimes inappropriate in concurrent ASD, due to restricted thinking and social interaction difficulties, as demonstrated here. There is hesitance to start LEF in psychiatric diagnoses, like ARFID, as the underlying issue is not tackled. However, ASD, a lifelong developmental disability, is often driving ARFID, so psychological interventions alone may be ineffective. This particularly applies to adults with ARFID as psychological interventions were found to be less effective than in children/adolescents. Therefore, in severe malnutrition despite intervention, LEF benefits likely outweigh the risks. Psychological support should continue whilst LEF improves nutrition, offering possible earlier discharge to an environment more conducive to improving oral intake and general mental health.

**Conclusion:** ASD-associated ARFID requires a different therapeutic approach to ARFID alone. Although the initial priority should be meeting nutritional requirements orally, LEF should be considered in severe individual cases to improve quality of life, reduce admissions and reduce mortality, but further research is required to improve outcomes.

